# A nomogram to predict skip metastasis in papillary thyroid cancer

**DOI:** 10.1186/s12957-020-01948-y

**Published:** 2020-07-15

**Authors:** Wenlong Wang, Zhi Yang, Qianhui Ouyang

**Affiliations:** 1 General Surgery Department, Xiangya Hospital, Central South University, No.87 Xiangya Road, Changsha, 410008 China; 2 Department of Colorectal & Anal Surgery, Hepatobiliary & Enteric Surgery Rearch Center, Xiangya Hospital, Central South University, No.87 Xiangya Road, Changsha, 410008 Hunan Province China

**Keywords:** Nomogram, Skip metastasis, Lymph node metastasis, papillary thyroid cancer

## Abstract

**Background:**

Skip metastases are defined as lateral lymph node metastasis (LNM) without the involvement of central LNM in papillary thyroid cancer (PTC), and it is difficult to predict in clinical practice. Our study aimed to investigate the risk factors of skip metastasis and establish a nomogram for predicting the probability of skip metastasis in PTC patients.

**Patients and methods:**

A total of 378 consecutive PTC patients with clinically suspected LNM who underwent modified radical neck dissection (MRND) from March 2018 to July 2019 in our hospital were enrolled. Univariate and multivariate analyses were used to examine risk factors of skip metastasis, and a nomogram prediction model was established and internally validated.

**Results:**

The incidence of skip metastases was 11.6% (44/378). Primary tumor size of ≤ 1 cm (OR = 2.703; 95% CI, 1.342–5.464; *P* = 0.005), age (OR = 1.051; 95% CI, 1.017–1.805; *P* = 0.005), and primary tumor location in the upper portion (OR = 6.799; 95% CI, 2.710–17.060; *P* < 0.001) were found to be independent risk factors for skip metastasis in PTC patients. A nomogram based upon these predictors performed well. The area under the curve (AUC) was 0.806 (95% CI, 0.736–0.876), and the *P* value of the Hosmer-Lemeshow goodness of fit test was 0.66. Decision curve analysis revealed that the nomogram was clinically useful.

**Conclusion:**

Based on the risk factors of skip metastasis, a high-performance nomogram was established, which can provide an individual risk assessment and can guide treatment decisions for patients.

## Introduction

The worldwide incidence of papillary thyroid carcinoma (PTC) has been steadily increasing in recent years [1]. In China, PTC is the most common malignant tumor in women under 30 years old, and its incidence rate ranks 8th in women [2, 3]. PTC often disseminates into cervical lymph nodes with a reported incidence from 30 to 80% at the first diagnosis [4, 5]. Cervical lymph node metastasis (LNM) is an important factor affecting local recurrence of PTC and long-term survival of patients [6, 7]. Generally, LNM of PTC occurs in a stepwise fashion, spreading from the thyroid lobes. LNM in PTC involves the central lymph node (CLN) first, then to the ipsilateral lateral lymph node (LLN), and finally arriving at the contralateral LLN and mediastinal compartment [8]. However, not all patients with PTC follow the drainage pathway for metastasis. Some patients develop lateral LNM without the involvement of central LNM via histopathological diagnosis, which is referred to as “skip metastasis” [6, 9, 10]. The rate of skip metastasis in PTC ranges from 1.6 to 21.8% [11–13]. The significance of skip metastasis in patients with PTC is still unclear; however, if untreated, the risk will increase for locoregional recurrence and distant metastasis. Reoperation and I^131^ therapy to treat locoregional recurrence or distant metastasis may affect patient’s quality of life. Thus, it is necessary for surgeons to identify the risk factors of skip metastasis before surgery and subsequent surgical intervention is important in preventing locoregional recurrence. In clinical work, the diagnosis of lateral neck LNM relies first on ultrasound, and fine needle aspiration cytology (FNAC), thyroglobulin, or BRAF-V600E detection are only recommended for suspicious LLN. However, ultrasound has a high specificity and low sensitivity in the diagnosis of cervical LNM [14, 15]. For patients with no enlarged lymph nodes in the central compartment before surgery, ultrasound doctors often relax their vigilance in the assessment of lateral lymph nodes and can easily omit to skip metastasis. Previous studies [13, 16–18] have reported that tumor located in the upper portion and with tumors diameter of no larger than 1 cm were closely linked to skip metastasis, and suggested that patients with the above risk factors should be carefully evaluated for lymph node status. Unfortunately, these studies did not give the probability of skip metastasis in these patients, or what kind of patients needed to undergo prophylactic LLN dissection.

To address this, our study first established an individual nomogram model for predicting skip metastasis. The nomogram is a practical and simple tool for identifying high-risk patients and quantifying individual risk, which has been frequently reported in cancer research [19–22]. The aim of this study was to develop a nomogram to predict skip metastasis in PTC patients to provide an individual risk assessment and guide treatment decisions for patients.

## Patients and methods

### Patients

This study retrospectively reviewed the clinical records of 401 patients who underwent modified radical neck dissection (MRND) from March 2018 to July 2019 in our hospital. Inclusion criteria were as follows: (I) PTC patients with complete medical records, (II) PTC patients with clinically suspected LNM preoperative who underwent MRND, and postoperative histopathology confirmed PTC with lateral LNM. The exclusion criteria for this study were as follows: (I) family history of thyroid cancer, (II) history of neck surgery, (III) patients who underwent iodine 131 before surgery, and (IV) tumor located in the isthmus. After strict inclusion and exclusion criteria, a total of 378 patients met the requirements. All patients knew and agreed to the treatment plan. Our study was approved by the Ethics Committee of Xiangya Hospital of Central South University.

### Surgical techniques

Ultrasound (US) and enhanced computed tomography (CT) were routinely performed in this study to assess the cervical lymph nodes and thyroid nodules before surgery. Fine needle aspiration biopsy (FNAB) was not systematically performed in our hospital. If the intraoperative frozen pathological examination was confirmed as PTC, prophylactic CLN dissection was conducted after total thyroidectomy. The CLN dissection ranged from superiorly by the hyoid bone, inferiorly to the sternal notch, and lateral to the carotid sheath, posteriorly to prevertebral fascia, including pretracheal, prelaryngeal (Delphian), perithyroidal, and paratracheal nodes. Lateral compartment includes levels II–IV. If lateral LNM was proven by FNAB or evident on preoperative US and enhanced CT, MRND was performed. While sparing the sternocleidomastoid muscle, internal jugular arteriovenous, spinal accessory nerve and other important structures, the lateral compartment was delimited inferiorly to the subclavian vein, superiorly to the sublingual nerve, and lateral to the anterior edge of the trapezius muscle. All specimens were sent to the Department of Pathology; the histopathological evaluation of these specimens was conducted by pathologists with at least 8 years of experience and who had diagnosed more than 400 PTC cases.

### Clinicopathological properties

Clinicopathological variables such as gender, age, primary tumor size, total tumor size, primary tumor location, tumor extension, multifocality, bilaterality, extrathyroidal extension (ETE), capsule invasion, Hashimoto’s thyroiditis (HT), number of central dissected lymph node, and number of lateral dissected lymph nodes involved. Primary tumor location was divided into three parts (upper, middle, and lower) based on the thyroid lobe involved. Primary tumor size was defined as the largest tumor in the specimen. Total tumor size means the sum of all tumor diameters in the specimen. Tumor extension was classified into four stages (T1, T2, T3, and T4) following the TNM Classification System for Differentiated Thyroid Carcinoma established by the American Joint Committee on Cancer (AJCC). Multifocality means two or more tumors lesion in the thyroid. Bilaterality is defined as the presence of carcinoma in both thyroid lobes. ETE was regarded as the tumor penetrating through the capsule and invading skeletal muscle tissue or perithyroidal soft tissue. In contrast, capsule invasion was defined as the tumor invading into the thyroid capsule, but not penetrating it. The HT patient was diagnosed by pathological examination.

### Statistical analyses

Statistical analysis was performed using the SPSS (19.0 version) and R software (version 3.4.2). All tests were two-sided, and *P* < 0.05 was considered statistically significant. Categorical variables were expressed as percentage (%) and frequency. Fisher’s exact test or chi-square test was used for categorical variables. Continuous variables were expressed as the mean ± SD and were compared using the *t* test or Mann-Whitney *U* test. Multivariate analysis was performed to screen for significant predictors of skip metastasis. The significant predictors were combined based on multivariate analysis, which was used to develop a nomogram. The nomogram model was calibrated using a calibration plot, and the Hosmer-Lemeshow goodness of fit test (*P* > 0.05), C-index values, and ROC curves were used to test its discrimination. The area under the curve (AUC) was calculated. The calibration plot with bootstrapping was used to illustrate the association between the predicted probability and actual probability. The clinical usefulness of the nomogram was evaluated using the decisions curve analysis.

## Results

### Patients’ characteristics

A total of 378 patients were enrolled in this study. There were 264 female and 114 male patients, and the ratio of female to male was 2.32:1. The age of the patients ranged from 10 to 72 years, with a mean age of 39.96 years. Additionally, 88.89% of patients were younger than 55 years old. Among all patients, 95 (25.13%) patients exhibited capsule invasion, and 69 (18.25%) patients presented ETE. Bilaterality was detected in 119 (30.95%) patients and HT was detected in 87 (23.16%) patients. Multifocal tumors (*n* = 84, 22.22%) were less common than solitary tumors (*n* = 294, 77.78%). Primary tumor location was divided into upper (*n* = 92), middle (*n* = 180), and lower portion (*n* = 106). According to the AJCC differentiated Thyroid Carcinoma guidelines, T1, T2, T3, and T4 were found in 219, 48, 83, and 28 patients, respectively. Primary tumor size of less than 1 cm was detected in 142 patients. The mean number of total central neck lymph nodes and lateral neck lymph nodes was 7.51 ± 5.08 and 18.25 ± 12.59, respectively (Table 1).

**Table 1:** Demographics and clinical characteristics of PTC patients (*n* = 378)

Variable	Results
Sex	
Male	114 (30.16%)
Female	264 (69.84%)
Age	
≧ 55 years	42 (11.11%)
< 55 years	336 (88.89%)
Age (mean ± SD, years)	39.96 ± 11.73
Multifocality	
Yes	84 (22.22%)
No	294 (77.78%)
Bilaterality	
Yes	117 (30.95%)
No	261 (69.05%)
Primary tumor location	
Upper	92 (24.34%)
Middle	180 (47.62%)
Lower	106 (28.04%)
HT	
Yes	87 (23.16%)
No	291 (76.98%)
ETE	
Yes	69(18.25%)
No	309(81.75%)
Capsule invasion	
Yes	95 (25.13%)
No	283(74.87%)
Tumor extension	
T1	219 (57.94%)
T2	48 (12.70%)
T3	83 (21.96%)
T4	28 (7.41%)
Primary tumor size ≦ 1 cm	
Yes	142 (37.57%)
No	236 (62.43%)
Total tumor size (mean ± SD, cm)	1.56 ± 0.96
Skip metastasis	
Yes	44(11.6%)
No	334(88.4%)
Number of central dissected lymph nodes	7.51 ± 5.08
Number of lateral dissected lymph nodes	18.25 ± 12.59

*HT* Hashimoto’s thyroiditis, *ETE* extrathyroidal extension, *SD* standard deviation

Among all patients, 44(11.6%) presented skip metastasis as lateral LNM without central LNM. The distributions of skip metastasis are shown in Table 2. Single-level metastasis (*n* = 20) was the most common pattern for lateral LNM, followed by double-level metastasis (*n* = 16), triple-level metastasis (*n* = 7), and four-level metastasis (*n* = 1). Further analysis showed that levels III and IV were the most involved sites, whether in single-level metastasis or double-level metastasis (Table 2).

**Table 2:** Distribution of skip metastasis

Neck level	No
Single level (*n* = 20)	
II	2
III	10
IV	8
Double level (*n* = 16)	
II + III	4
II + IV	1
III + IV	9
III + V	1
IV + V	1
Triple level (*n* = 7)	
II + III + IV	4
III + IV + V	3
Four level (*n* = 1)	
II + III + IV + V	1
Total	45

### Clinicopathologic risk factors for skip metastasis

Univariate analysis demonstrated that age, primary tumor location, and primary tumor size ≤ 1 cm were associated with skip metastasis (all *P* < 0.05). Patients with skip metastasis were significantly older than patients without (44.48 ± 12.556 vs. 39.37 ± 11.509, *P* = 0.006). When the primary tumor was located in the superior, middle, or inferior portion of the thyroid, the incidence of skip metastasis was 7.41%, 2.38%, and 1.85%, respectively. In addition, a primary tumor size not larger than 1 cm was more frequent in skip metastasis (54.55% vs. 35.33%, *P* = 0.013; Table 3).

**Table 3:** Univariate analysis of risk factors for skip metastasis in PTC patients

Variable	Skip metastasis		*χ*^2^/*t*	*P* value
	Present (*n* = 44)	Absent (*n* = 334)		
Sex (male/female)	9/35	105/229	2.23	0.136
Age(≧ 55 years/< 55 years)	8/36	34/300	2.52	O.112
Age	44.48 ± 12.56	39.37 ± 11.51	2.74	0.006
Multifocality (yes/no)	7/37	77/257	1.15	0.284
Bilaterality (yes/no)	16/28	101/233	0.68	0.409
Primary tumor location upper/middle/lower	28/9/7	64/171/99	41.93	< 0.001
HT (yes/no)	9/35	78/256	0.18	0.668
ETE (yes/no)	8/36	61/273	0.001	0.989
Capsule invasion (yes/no)	11/33	84/258	0.001	0.983
Tumor extension (T1/T2/T3/T4)	23/7/12/2	196/41/71/26	1.87	0.6
Primary tumor size ≦ 1 cm (yes/no)	24/20	118/216	6.12	0.013
Total tumor size/cm	1.31 ± 0.87	1.59 ± 0.97	1.79	0.075
Number of central dissected lymph nodes	6.47 ± 4.59	7.65 ± 5.14	1.46	0.144
Number of lateral dissected lymph nodes	16.89 ± 10.12	18.44.47 ± 12.98	0.77	0.439

*HT* Hashimoto’s thyroiditis, *ETE* extrathyroidal extension

To identify the independent risk factors of skip metastasis in PTC patients, variables with statistical differences were incorporated in a multivariate analysis. We found that primary tumor size ≤ 1 cm (OR = 2.703, 95% CI, 1342–5.464; *P* = 0.005), primary tumor location in the upper portion (OR = 6.799, 95% CI, 2.710–17.060; *P* < 0.001), and age (OR = 1.051, 95% CI, 1.017–1.805; *P* = 0.005) were found to be independent factors for skip metastasis in PTC patients (Table 4).

**Table 4:** Multivariate analysis of risk factors for skip metastasis in PTC patients

Variable	OR (95% CI)	*P* value
Age	1.051 (1.017–1.805)	0.003
Primary tumor location		
Lower as reference		
Middle	0.678 (0.241–1.912)	0.463
Upper	6.799 (2.710–17.060)	< 0.001
Primary tumor size ≦ 1 cm (yes/no)	2.703 (1.342–5.464)	0.005

### Construction of an individualized prediction model

**Fig. 1:**
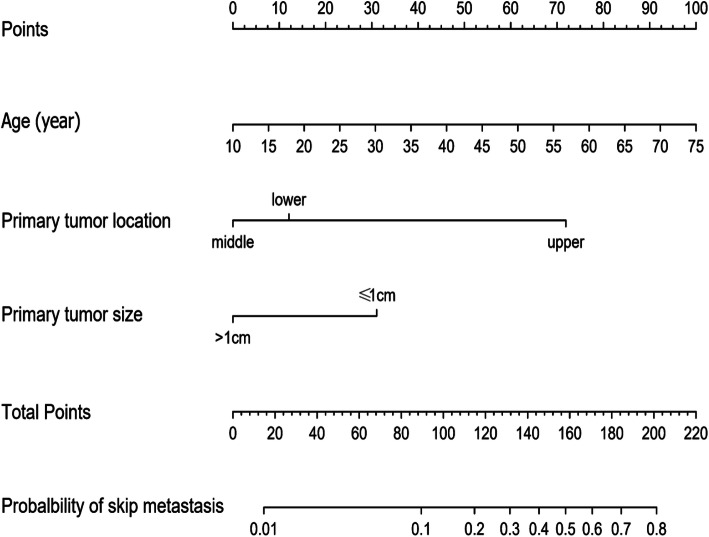
Nomogram predicting the probability of skip metastasis

Based on the results of the multivariate analysis, we established a nomogram model for predicting skip metastasis (Fig. 1) that can predict the probability of skip metastasis in patients by summing the scores of each variable. Older patients, primary tumors location in the upper portion and primary tumor sizes ≤ 1 cm had higher scores. For example, a 55-year-old (69 points) PTC patient with a 7-mm tumor (31 points) located in the upper portion (72 points), has a probability of skip metastasis of about 60% (172 total points). The risk of skip metastasis predicted by this nomogram ranged from 0.01 to 0.8.

### Model performance and clinical utility of the nomogram

**Fig. 2:**
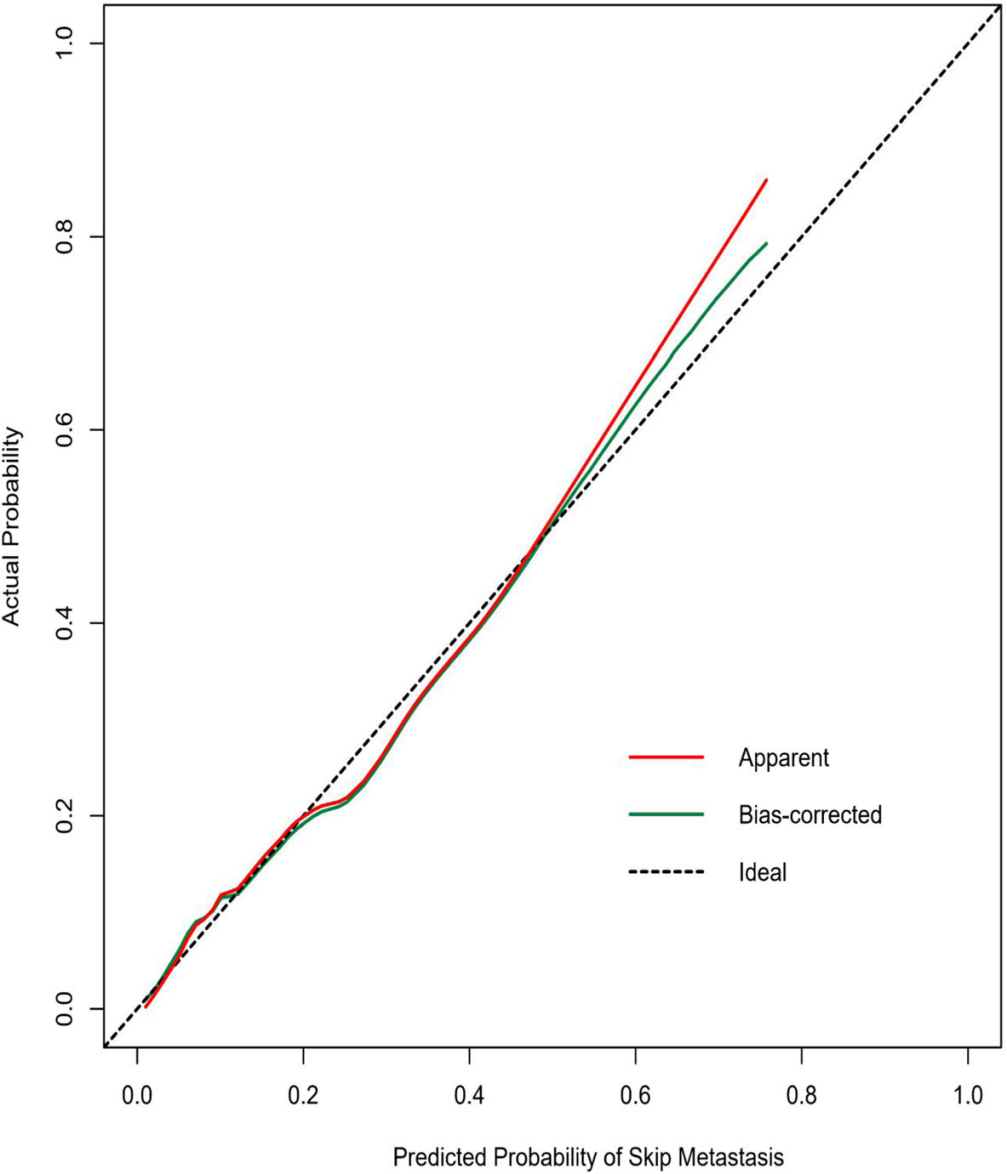
Calibration curves of the nomogram for the probability of skip metastasis. On the calibration, the *y*-axis represents the actual probability; the *x*-axis represents the nomogram-predicted probability of skip metastasis. The dotted black line is the ideal curve; the blue line represents the bias-corrected curve, and the red line represents the nomogram

**Fig. 3:**
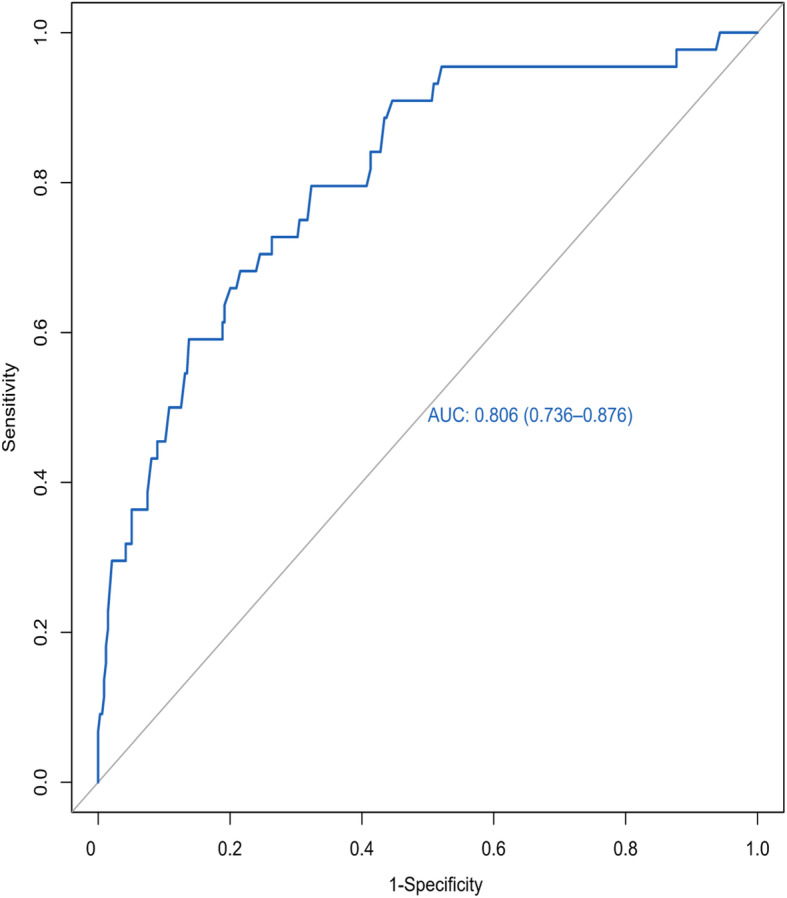
The ROC curve of nomograms for skip metastasis. The area under the ROC curve (AUC) is 0.806, 95% CI 0.736–0.876. ROC receiver operating characteristic, AUC area under curve

**Fig. 4:**
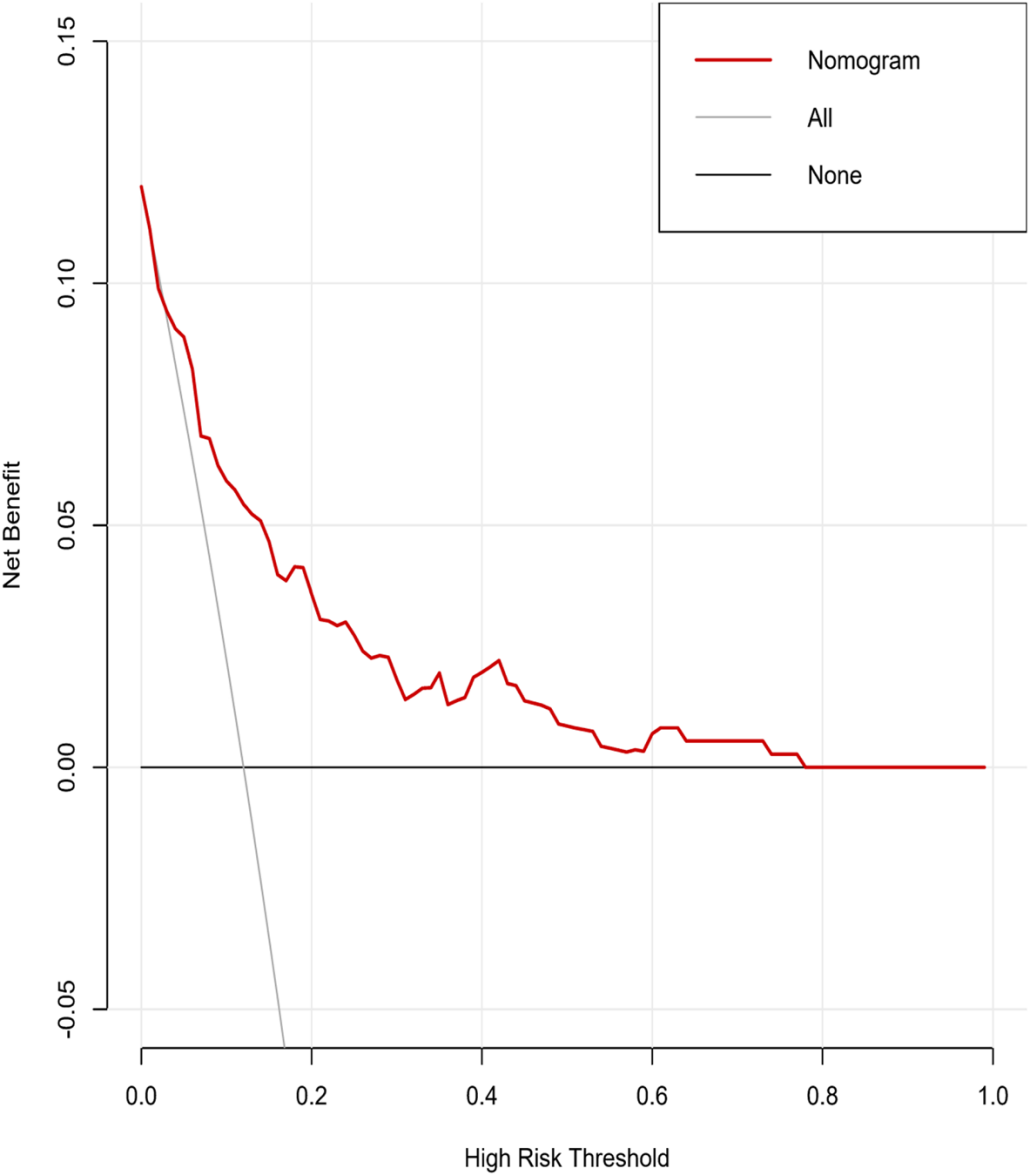
Decision curve analysis for nomogram. The black line represents the hypothesis that all PTC patients do not have skip metastasis. The gray line represents the hypothesis that all patients with PTC present skip metastasis. The red line represents the nomogram. The *y*-axis represents net benefit, and the *x*-axis represents threshold probability

To test its consistency and discrimination, the nomogram model was calibrated by Hosmer-Lemeshow goodness of fit test and calibration plot. The internal calibration plot showed a mostly perfect agreement between the predicted and actual results of the nomogram model, as shown in Fig. 2. The Hosmer-Lemeshow goodness of fit test also showed an excellent concordance between the predicted and actual outcomes (*χ*^2^ = 5.89, df = 8, *P* = 0.66). ROC curves and C-index values were used to test the discrimination of the nomogram model. The C-index value was 0.806 and the area under the curve (AUC) was 0.806 (95% CI, 0.736–0.876), indicating good discrimination of the model (Fig. 3). In the decision curve analysis (DCA) curve, when the skip metastasis of threshold probability ranged from 0.04 to 0.78, the nomogram model achieved a greater net benefit than the “None” or “All” (Fig. 4).

Nomograms show the likelihood of skip metastasis as a percentage, and we assigned a Youden-derived cutoff value to the nomogram. The optimal cutoff value was − 2.374 (sensitivity, 79.5%; specificity, 67.7%; accuracy, 69.0%; negative predictive value, 96.2%; positive predictive value, 24.5%).

## Discussion

Cervical LNM is common in PTC patients and accounts for 30–80% [4, 5, 21]. LNM was associated with local recurrence of PTC and overall mortality of patients [6, 7]. Reoperation for PTC recurrence may increase operative complications and have a negative effect on a patient’s quality of life. Thus, it is important for surgeons to perform precise preoperative evaluations and predictions for LNM to take an appropriate surgical management strategy. “Skip metastasis” is defined as lateral LNM without the involvement of central LNM, which is not uncommon in clinical practice, yet is difficult to predict [11, 13, 16, 22, 23]. In this study, we developed an individualized nomogram to evaluate the likelihood of skip metastasis in patients with PTC based on the pathologic and clinical characteristics, which is consistent with the current trend toward personalized and precision medicine.

The incidence of skip metastasis in the literature ranges from 1.6 to 21.8%, which could be explained by different religions and sample sizes [15, 24]. However, previous studies were limited by low patient numbers and a heterogeneous patient population [10, 14, 22]. Our study used a sufficient number of patients (*n* = 378), and the rate of skip metastasis was 11.6%, which is in accordance with previous study.

The mechanism of skip metastasis is still unclear. Machens et al. [22] summarized the clinical data of 13 patients with skip metastasis of PTC and proposed that skip metastasis is an unstable lymph node metastasis phenomenon of thyroid cancer that is a rare and occasional metastatic mode and is not due to limited or missed lymph node samples. Skip metastases most frequently showed single-level metastases in the lateral compartment and less multiple-level metastasis, and level III nodes were the most frequently involved sites, followed by levels IV, II, and V [14–16].

Next, we explored the predictive factors associated with skip metastasis in PTC. In the univariate analyses, we found that age, primary tumor location, and primary tumor size ≤ 1 cm were significantly related to skip metastasis in PTC patients (all *P* < 0.05). These variables were also the independent predictors of skip metastasis, and their predictive value was verified by multivariate analysis, while gender, total tumor size, tumor extension, multifocality, bilaterality, ETE, HT, and capsule invasion were not significantly associated with skip metastasis (all *P* > 0.05; Tables 2 and 3). In this study, patients with primary tumor location in the upper portion were more likely to have skip metastasis, which is in line with previous reports [13, 16, 24]. This predictive factor could be explained by the anatomical structure of the lymphatic drainage system. However, Lim et al. [10] reported that the primary tumor location in the upper pole was inversely correlated with skip metastasis. The relationship between skip metastasis and tumor location remains controversial and needs further research. In addition, a primary tumor size not larger than 1 cm was more frequent in skip metastasis (54.55% vs. 35.33%, *P* = 0.013). These results remind us that the primary tumor size is an important factor for predicting skip metastasis, and clinicians should carefully evaluate lateral lymph node status when tumor sizes are no larger than 1 cm. Previous studies also revealed that skip metastasis was more common in less aggressive forms of PTC [16]. Furthermore, this study demonstrated that the risk of skip metastasis is associated with age, and increasing age was significantly related to an increased risk of skip metastasis (44.48 ± 12.556 vs. 39.37 ± 11.509, *P* = 0.006). This finding has not been reported in previous studies. According to the 8th American Joint Committee on Cancer (AJCC) TNM staging system [25], the age of 55 years old was the cutoff value, and patients aged ≥ 55 years old could have higher risk factors for LNM. However, we did not find that age of more than 55 years was related to skip metastasis.

Based on the abovementioned significant factors related to skip metastasis in PTC, a predictive nomogram was constructed. To date, there is no report on the use of a nomogram to predict skip metastasis. This study summarized clinical data and first established a nomogram that performed well in the prediction of skip metastasis. The calibration plots showed good agreement between predicted probability and actual probability of skip metastasis. Likewise, the AUC of the nomogram in this study was 0.806 (95% CI, 0.736–0.876); according to previous studies, an AUC value greater than 0.7 has superior accuracy, indicating good discrimination. Clinical decision curve analysis demonstrated that most PTC patients could benefit from the predictive model. Thus, utilization of this nomogram can provide an individual risk assessment and guide treatment decisions for patients. With an optimal cutoff value, the nomogram yielded 79.5% sensitivity, 67.7% specificity, and the maximum at the probability of skip metastasis was 0.085, corresponding to a total point value of 83.04. Therefore, patients with a total point value of more than 83.04 are considered to be high risk for skip metastasis, and MRND should be considered. However, for patients with a total point value of less than 83.04, close observation with US and follow-up are recommended. Thus, we confirmed that our nomogram was an objective and useful tool to aid clinicians in deciding whether to perform MRND rather than making a decision based on rough and simple clinicopathological characteristics.

Our nomogram has several limitations. First, this study was a retrospective, single-center study, which may result in selection bias and information bias; therefore, the dataset cannot represent the whole PTC population. Second, the validation of our nomogram was only conducted internally, and external validation should be performed to ensure that it has better extrapolation. Third, although our nomogram may identify patients at high risk of skip metastasis, whether undergoing MRND is beneficial for improving long survival is still unknown. Besides, the US is limitation in central lymph node detection, and thus some skip metastases may be wrong. Because central and ipsilateral neck nodes are equally involved, a negative US result in both the lateral and the central neck cannot reliably exclude lymph node metastasis, rendering it extremely challenging to ascertain false negative rates reliably, which are in the order of 25–59% in the lateral and 44–74% in the central neck [26]. Despite these shortcomings, our nomogram was based on reliable clinical data obtained from patients with satisfactory manifestations of good discriminative ability and internal validation.

## Conclusion

In conclusion, we developed a predictive nomogram for skip metastasis in PTC patients, which can help identify patients at high risk of skip metastasis who need to undergo MRND. Thus, utilization of this nomogram can provide an individual risk assessment and guide treatment decisions for patients.

## Data Availability

The datasets used and/or analyzed during the current study are available from the corresponding author on reasonable request.

## Abbreviations

LNM: Lateral lymph node metastasis
PTC: Papillary thyroid cancer
CLN: Central lymph node
LLN: Lateral lymph node
US: Ultrasound
CT: Enhanced computed tomography
MRND: Modified radical neck dissection
HT: Hashimoto’s thyroiditis
ETE: Extrathyroidal extension
SD: Standard deviation
ROC: Receiver operating characteristic
AUC: Area under curve
DCA: Decision curve analysis
AJCC: American Joint Committee on Cancer
